# An SDS-NaOH-based method to isolate genome of recombinant adeno-associated virus vectors for physical titer measurement

**DOI:** 10.1371/journal.pone.0315921

**Published:** 2025-04-03

**Authors:** Xiangying Zhu, Keying Yang, Jinyan Xie, Xilin Feng, Tao Wu, Mengjun Hu, Haijian Wang, Chenghui Yu, Xiaomin Yu, Farhid Hemmatzadeh, Liqing Zhu, Linhua Zhang

**Affiliations:** 1 Zhejiang Hengyu Biological Technology Co., Ltd., Jiaxing, Zhejiang, China; 2 State Key Laboratory of Genetic Engineering and Engineering Research Center of Gene Technology (Ministry of Education), School of Life Sciences, Fudan University, Shanghai, China; 3 Department of Clinical Laboratory, The First Affiliated Hospital of Wenzhou Medical University, Wenzhou, Zhejiang, China; 4 School of Animal and Veterinary Sciences, The University of Adelaide, Adelaide, South Australia, Australia; 5 Department of Clinical Laboratory, Peking University Cancer Hospital and Institute, Beijing, China; 6 Department of Clinical Laboratory, The People’s Hospital of Yuhuan, Taizhou, Zhejiang, China; CNR, ITALY

## Abstract

Recombinant adeno-associated viruses (rAAVs) vectors are promising for their safety and sustained expression of genetic payloads across various tissues. These vectors consist of a protein capsid enclosing a 4.7 kb single-stranded DNA genome. Rapid and accurate determination of the physical titers of rAAV vector is crucial for quality control in rAAV manufacturing and precise drug dosage in clinical trials. To prepare vector DNA for genome titer assessment, it is essential to completely degrade unencapsulated DNA and dissociate the capsid. Conventional methods typically involve co-incubation with DNase I to degrade unencapsidated DNA, followed by co-incubation with Proteinase K to cleave protein shells. Here, we present a “Benzonase & SDS-NaOH" pretreatment as an effective alkaline lysis for releasing the vector DNA. In the presence of producer cell crude extract, Benzonase demonstrated superior efficacy in degrading unencapsidated DNA compared to DNase I. Additionally, the use of SDS-NaOH, effective at 65 °C for 30 min, significantly reduces the time required compared to that of Proteinase K at 56 °C for 2 hours. We also showed that the “Benzonase & SDS-NaOH" pretreatment is applicable for vector genome titration in rAAV production, harvest, and purified stock. Moreover, our method is effective for both scAAV and ssAAV forms and across all serotypes, including the thermally stable rAAV5. Overall, this method offers a rapid and straightforward solution to determine rAAV vector genome titers in both purified preparations and during the manufacturing process.

## Introduction

The recombinant adeno-associated virus (rAAV) vector, derived from wild-type AAV and optimized through genetic engineering, has been an ideal gene delivery vector for decades [1,2]. Renowned for its long-term transgene persistence in vivo and broad host cell tropism with minimal toxicity, rAAV vectors have gained popularity as biotherapeutic agents [3,4]. To date, rAAV-based biologics have been successfully marketed: Upstaza [5], for aromatic L-amino acid decarboxylase (AADC) deficiency; Roctavian [6], for hemophilia A patients; Luxturna [7], for inherited retinal disease (IRD); Zolgensma [8], for spinal muscular atrophy (SMA); and Hemgenix (EtranaDez) [9], for hemophilia B. Beyond North America and Europe, where these drugs are commonly marketed, rAAV-based therapies are becoming increasingly popular in research, and several are currently undergoing clinical trials in China [10,11]. Notably, NR082 [12] targets Leber’s hereditary optic neuropathy (LHON) caused by ND4 mitochondrial gene mutation; BBM-H901 [13] delivers hFIX Padua cDNA to treat Hemophilia B; and LX101 [14] employs rAAV2-RPE65 to cure RPE65-associated inherited retinal degeneration. Prior to the administration of rAAV drugs in clinical trials, precise quantification of vector genome (vg) must be determined. There is a linear correlation between transgene expression and the administered dose of rAAV. However, medical incidents involving rAAV-FIX overdose occurred due to underestimation of rAAV vg titers. The reason is believed to be primer mismatches within the inverted terminal repeat (ITR) region in the self-complementary (sc) rAAV genomes [15,16]. Therefore, accurate vg titration is essential for precise dosing and effective treatment [8].

Several methods have been employed to determine rAAV vg titers, including Slot-blot [17], quantitative real-time PCR (qPCR) [18,19], digital droplet PCR (ddPCR) [20], Southern Blot [17], DNA-staining with agarose gel [21], and high-throughput techniques such as high-performance liquid chromatography (HPLC) [22], analytical ultracentrifugation (AUC) [23], and UV spectrophotometry [24]. Each method has distinct strengths and limitations. Currently, PCR-based techniques, particularly qPCR, are widely favored due to their robustness, accuracy, and specificity [25,26]. While ddPCR is an emerging method with potential, its high cost limits its practicality for large-scale industrial applications. Additionally, qPCR offers a broader dynamic range than ddPCR, enabling the detection of nucleic acid molecules over a wide concentration range. As such, qPCR is the standard method for rAAV vg titer determination in both research and industry settings.

In rAAV vg titration, it is essential to remove non-encapsulated DNAs to avoid interfering with accurate measurements [25]. To this end, DNase I is a well-established and widely implemented method in both research and industry settings [18,27]. The duration of DNase I treatment can vary significantly, ranging from 15 min to overnight at 37 °C. Alternatively, Benzonase [28], a modified endonuclease from *Serratia marcescens*, has been proposed as a substitute for DNase I [27]. Benzonase effectively cleaves both DNA and RNA, regardless of whether they are single-stranded, double-stranded, linear, or circular [29]. In addition, Benzonase exhibits higher catalytic efficiency compared to DNase I. Following nucleic acids removal encapsidated vector DNA can be released from denatured capsid proteins using three main methods: thermal lysis, enzymatic cleavage, and alkaline lysis. Thermal lysis typically involves incubating samples at 95 °C for at least 10 min. However, it is not suitable for all rAAV serotypes, particularly those with high heat resistance, such as rAAV1 and rAAV5, and therefore is not widely adopted [30,31]. Enzymatic cleavage generally refers to adding Proteinase K, followed by a heating cycle at 50 °C for 60 min, then at 95 °C for 10 min, and a subsequent cooling at 4 °C [32]. The alkaline lysis method employs NaOH to denature the protein shell, often combined with thermal lysis, such as incubating rAAV with NaOH at 80 °C for 10 min [33]. Despite the widespread use of the above pretreatments before qPCR assays, there has been no comprehensive comparison of their efficacy at various stages of the rAAV production process.

**Fig 1:**
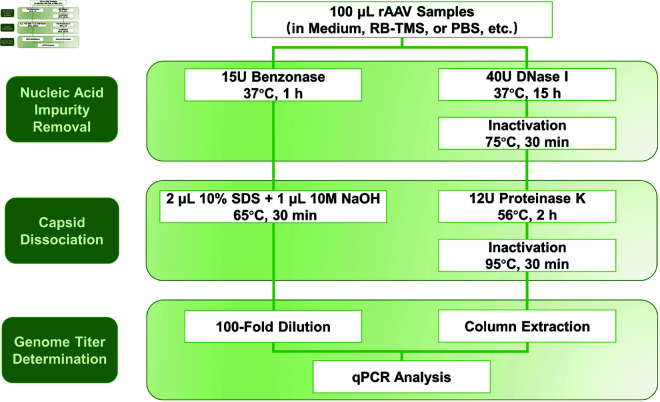
Flowchart of two pretreatments for qPCR determination of rAAV vg titer. The 100 *μ*L rAAV sample consists of 10 *μ*L of rAAV crude lysate or purified stock, with the remaining 90 *μ*L comprising the buffer. In the “Benzonase & SDS-NaOH" pretreatment, 10 *μ*L of vector was incubated with 0.5 *μ*L (15U) of Benzonase (70664-4CN, Merck, US) at 37 °C for 1 h. Then, 2 *μ*L of 10% SDS and 1 *μ*L of 10M NaOH were added and the mixture was further incubated at 65 °C for 30 min. In the “DNase I & Proteinase K" pretreatment, 10 *μ*L of vector was treated with 4 *μ*L (40U) of RNase-free recombinant DNase (2270A, TAKARA, Japan) at 37 °C for 15 h. After inactivating DNase I at 75 °C for 30 min, 20 *μ*L of proteinase K (20 mg/mL) was added and the mixture was further incubated at 56 °C for 2 h, followed by inactivation at 95 °C for 30 min. For further purification of vector DNA, spin columns (DNA Clean & Concentrator-25, D4034, ZYMO Research, US) were used.

Here, we establish a simple and reliable “Benzonase & SDS-NaOH" pretreatment for quantifying rAAV vg titer, with a low LOD (Limit of Detection) and LOQ (Limit of Quantification). By a thorough comparison with the previously-used “DNase I & Proteinase K" pretreatment (Fig 1), we propose that this method can be broadly applied to rAAV vector quantification, potentially enhancing quality control of rAAV drug production in both large-scale industrial settings and small-scale laboratory tests.

## Materials and methods

### Cell culture

HEK293 cells (CRL-1573, ATCC, US) were maintained in DMEM (319-005-CL, WISTENT, China) supplemented with 10% fetal bovine serum (FBS) (WISTENT, Nanjing, China) and 1% Penicillin-streptomycin (086-550, WISTENT, China). Cell transductions were performed in DMEM with no serum. All the materials and equipment for cell culture and cell assays were listed in S1 Table.

### rAAV production and purification

**Vector production and harvesting.** Large-scale production of rAAVs of different serotypes including AAV2, AAV3, AAV5, AAV6, AAV8, and AAV9 was carried out by triple-transfection with: (1) cis plasmids containing the gene of interest driven by cytomegalovirus (CMV) promotor; (2) packaging plasmids with different capsid serotypes(e.g., pACG2); (3) adenovirus helper plasmid (pHelper). Each HEK293 culture plate (150-mm diameter) was co-transfected by PEI with a total of 60 *μ*g plasmids at a weight ratio of 1:1:2.

After 6 h of transfection, the medium on each plate was replaced with 25 mL of complete DMEM. The cells were pelleted 72 h post-transfection and resuspended in RB-TMS (50 mM Tris-HCl + 150 mM NaCl, pH = 8.0, Asepsis). The resuspended cells were subjected to three freeze-thaw cycles to lysis the cells and release the vectors within. The medium containing rAAV vectors was collected 72 h post-transfection and mixed with one-fourth of its volume of PEG 8000 (MB2594, Meilunbio, China) and shaked overnight at 4 °C to isolate the containing rAAV vectors, as PEG 8000 facilitates the sedimentation of rAAV. The PEG 8000-rAAV precipitate was combined with the mixture after three freeze-thaw cycles for further purification.

**Vector purification and concentration.** At the next step, 1 *μ*L of 4.8 M MgCl2, and 2 *μ*L (60 Units(U)) of Benzonase (70664-4CN, Merck, US) were added to combination and incubated at 37 °C for 30 min in a water bath to degrade cellular DNA debris. The mixture was purified by iodixanol gradient ultracentrifugation, following standardized procedures described previously [34]. The iodixanol-rAAV mixture phcollected was added to 25 mL of detergent (Buffer A). The ion exchange column chromatography was used to further purify the rAAV vectors using a 5-mL HiTrap Q HP column (17115301, GE Healthcare, US). The columns were sequentially rinsed with detergent, balance buffer (Buffer B), and enrichment solution (Buffer C), following the sample solution mentioned in S2 Table. The final rarefied sample was collected in eluent into a clean tube, which would later be concentrated by ultracentrifugation. The mixture obtained after the aforementioned steps is referred to as rAAV crude lysate, which can also be used for direct infection of cells.

The composition of required solutions in rAAV production and purification, including cell culture media, transfection reagents, purification, and concentration buffers, was listed in S2 Table.

### VG extraction

rAAV vector samples include crude lysates prepared in our lab using the triple-transfection method, and purified vectors produced under standard manual protocols or purchased from ATCC, the rAAV2-RSS (VR-1616, ATCC, US).

The “Benzonase & SDS-NaOH” pretreatment used a protocol included incubation with Benzonase (70664-4CN, Merck, US) and SDS-NaOH. Then, 10 *μ*L of the vector was treated with 0.5 *μ*L (15 Units (U)) Benzonase at 37 °C for 1 h according to the manufacturer’s instructions, to remove the residual plasmid DNA in vector samples. After incubation, 2 *μ*L 10% SDS and 1 *μ*L 10M NaOH were then added and incubated at 65 °C for 30 min to release vector DNA from AAV capsids.

The “DNase I & Proteinase K” pretreatment used a protocol incorporating incubation with DNase I and Proteinase K. 10 *μ*L of vector was treated with 4 *μ*L (40U) recombinant DNase (RNase free) (2270A, TAKARA, Japan) at 37 °C for 15 h to remove the residual plasmid DNA in vector samples. After the inactivation of DNase I at 75 °C for 30 min, 20 *μ*L of proteinase K (20 mg/mL) was then added and incubated at 56 °C for 2 h to release vector DNA from AAV capsids before being inactivated at 95 °C for 30 min [32].

After the pre-treatment described earlier, 100 *μ*L of a mixture, containing 10 *μ*L of rAAV sample, will be loaded onto the column. The vector DNA was extracted and purified using the column based extraction kit (DNA Clean & Concentrator-25, ZYMO 137 Research, US), under the guidance of the manufacturer’s instructions. This step is designed to eliminate any potential qPCR inhibitors and to determine whether DNA extraction is necessary for accurate rAAV vg titration.

### qPCR titration

Vg titers were quantified by SYBR Green qPCR in Tsingke 2× qPCR SYBR Green I mix (TSE203, Tsingke, China). pTR-UF11 (157970, Addgene, US) is a plasmid containing two recombinant AAV2 ITRs which serves as the standard for qPCR detection of rAAV vg titers. Standard curves were obtained using 2 × 10^3^ to 2 × 10^9^ copies per *μ*ŁpTR-UF11 plasmid. Primers were targeted to the ITRs.

ITR-Primer-F: 5’-GGAACCCCTAGTGATGGAGTT-3’ ITR-Primer-R: 5’-CGGCCTCAGTGAGCGA-3’

Components of each qPCR reaction and protocol for SYBR-Green-based qPCR analysis were listed as S3 Table and S4 Table. Vg titers were calculated from at least 3 independent titrations, and standard curve repetitions were reported in Table 1.

**Table 1:** Standard curve repeatability.

Standard	Best-fit values				Goodness of fit		Amplification factor	
**Curve Repeatability**	**Slope**	**Y-intercept when X = 0.0**	**X-intercept when Y = 0.0**	**1/slope**	**R square**	**Equation**	**qPCR Efficiency**	**Cq of NTC**
*Repeat*1	–3.3346	44.95	13.4798	–0.2999	0.9981	Y = –3.3346X + 44.95	99.47	35
*Repeat*2	–3.3275	44.87	13.4845	–0.3005	0.9981	Y = –3.3275X + 44.87	99.77	34.26
*Repeat*3	–3.2914	44.574	13.5425	–0.3038	0.9987	Y = –3.2914X + 44.574	101.29	35.12

### rAAV vectors transduction analyzed by fluorescence microscopy

HEK293 cells were seeded in 96-well cell culture plates at a density of 90%. Overnight, the appropriate amount of the vectors was diluted with DMEM without serum and added to the cell plate after supernatant removal at a total volume of 20 *μ*L. Then 100 *μ*L DMEM with 10% FBS was added after 2 h of transduction. Gene expression was detected by a fluorescence microscope. ImageJ was used to assess the total area of green fluorescence (pixel^2^) per visual field.

### GFP expression by flow cytometry

HEK293 cells were seeded in 12-well cell culture plates at a density of 5 × 10^5 ^cells/well. Overnight, the appropriate amount of the vectors was diluted with DMEM without serum and added to the cell plate after supernatant removal at a total volume of 200 *μ*L. Then 1 mL DMEM with 10% FBS was added after 2 h of transduction. Cells were harvested 72 h post-transduction, fixed with 10% FBS in DMEM, and resuspended in PBS. Analysis of GFP expression was performed on 10000 cells from each well in a BD FACSCabilur (FACSCabilur, BD Biosciences, US). FlowJo software was used to calculate the % GFP-positive cells.

### Statistical analyses

Data were represented as the group mean ± standard deviation (SD). Statistics were tested using Prism v.8.0 (Prism, GraphPad Software, US), using different statistical methods depending on the dataset to be analyzed and the experimental setup.

## Results

### 1. Benzonase digestion is recommended as a sample pre-treatment

The efficacy of Benzonase and DNase I in digesting nucleic acid impurities was compared using 1 *μ*g of plasmid DNA (pTR-UF11) and 1 *μ*g of HEK293 RNA, each treated with 15 U of the respective enzyme. S1A Fig shows that both enzymes effectively degraded DNA, but DNase I did not degrade RNA. In the next step, the enzymes’ performance in cell lysate were assessed by adding 1 *μ*g of pTR-UF11 to 10 *μ*L of HEK293 lysate and treated with Benzonase or DNase I. As shown in S1B Fig, 15 U of DNase I at 37 °C for 1 h did not completely digest the plasmid, while Benzonase achieved complete digestion.

**Fig 2:**
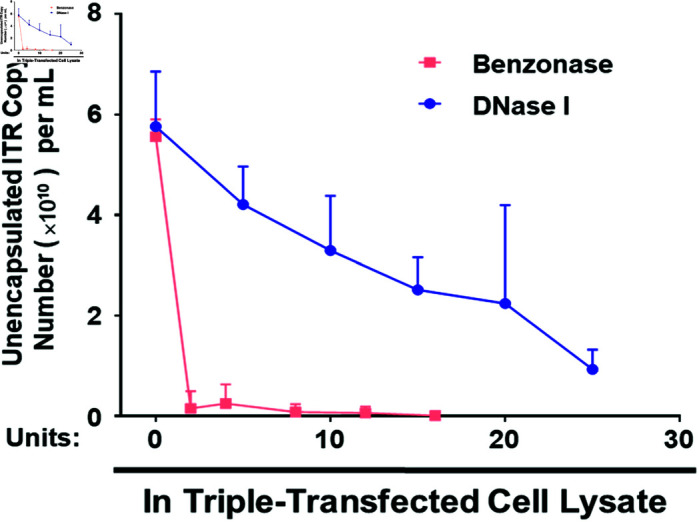
Benzonase has superior DNA degradation capacity than DNase I when digesting DNA impurities in rAAV crude lysate. HEK293 cells were triple-transfected with plasmids pACG2, pAAV-CMVp-*gfp* and pHelper. Crude cell lysates were obtained 72 h post-transfectoin, 10 *μ*L of which was mixed with 90 *μ*L of ddH_2_O and increasing concentrations of Benzonase (Red) or DNase I (Blue) at 37 °C for 1 h. Samples were subjected to qPCR assays against ITR to determine unencapsulated DNA copies. Graph bars represent means ± SD of n = 4 experiments.

**Fig 3:**
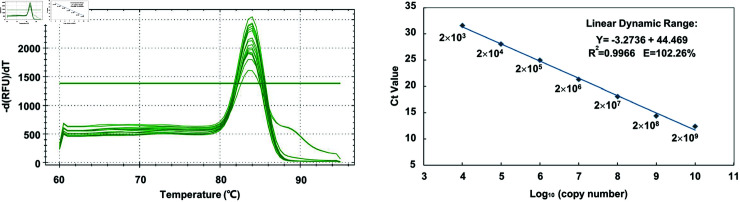
Performance of standard curve. Standard curve for SYBR-green RT-qPCR amplification of standard sample (pTR-UF11 plasmid). Amplification plots showing the testing in triplicate of a 10-fold dilution series containing pTR-UF11 ranging from 2× 10^9^ to 2× 10^13^ copies per microliter

To further determine the optimal concentration of Benzonase and DNase I for digesting unencapsidated DNA, HEK293 cells were triple-transfected to produce rAAV2-CMVp-*gfp* vectors. The crude cell lysates, collected 72 h post-transfection, were treated with increasing concentrations of Benzonase or DNase I. The samples, without disruption of the capsid protein, were analyzed using SYBR green-based qPCR with primers located at the ITR. It was evident that less than 5 U of Benzonase maximally eliminated unencapsulated ITR-containing DNA. In contrast, this concentration of DNase I was insufficient for complete digestion, leaving approximately 4 × 10^10^ copies of unencapsulated DNA after 1 h of digestion (Fig 2). In conclusion, Benzonase exhibits superior DNA digestion capabilities compared to DNase I in the presence of cell lysate, and a significantly higher quantity of DNase I is required to achieve equivalent DNA degradation. Performance testing of ITR primers was conducted to confirm their efficacy in amplifying and detecting the plasmid DNA pTR-UF11. Cycle threshold (Ct) values were obtained and extrapolated onto a calibration curve generated through the amplification of serial dilutions of pTR-UF11 using qPCR assays (Fig 3). The Ct values were plotted against known copy numbers of the standard sample, and the resulting standard curve demonstrated a linear range spanning seven orders of magnitude. The efficiency of the standard curve, ranging from 99% to 102% (Table 1), attests to the reliability of the qPCR assay. The slope of the standard curve (–3.2736) and its high correlation coefficient (R^2^ = 0.9966) confirm that this assay is suitable for the quantification of ITR-containing DNA.

### 2. Release of vector DNA by SDS-NaOH or proteinase K

**Fig 4:**
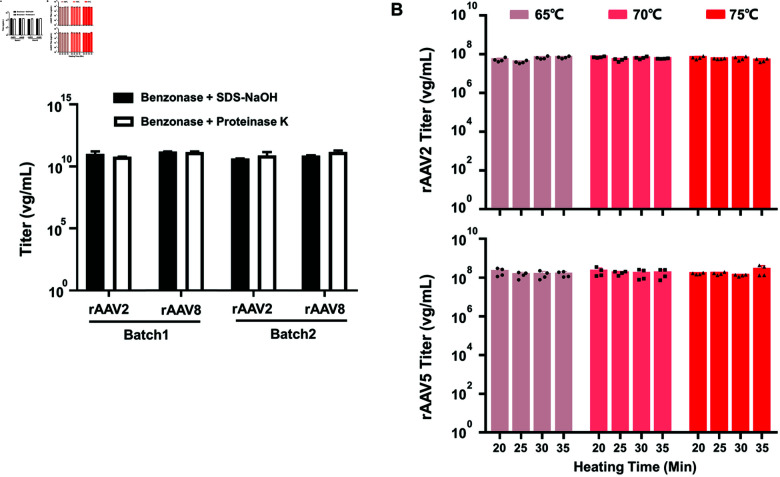
The efficiency of SDS & NaOH method and Proteinase K method for rAAV vector genome titer. A: Purified rAAV2- and rAAV8-CMVp-*gfp* vectors were produced in 2 batches and pre-digested by Benzonase. Samples were incubated with SDS-NaOH or Proteinase K, followed by detection of vg titer using qPCR analysis. B: Purified rAAV2 and rAAV5-CMVp-*gfp* vectors were diluted 1000-fold and pre-digested by Benzonase and then incubated with SDS-NaOH at varying temperatures and time periods, followed by the determination of vg titer using qPCR analysis. Statistical analysis was performed by unpaired t-test. DNA isolations have been conducted 4 times. Graph bars represent means ± SD of n = 4 experiments. No significant difference was observed between groups in these experiments. Titers were shown in the graph as Log10.

The release of vector DNA from the capsid is crucial for accurate rAAV titration. We evaluated two methods for this purpose: alkaline lysis using SDS-NaOH and enzymatic cleavage with Proteinase K. The alkaline lysis method involves heating samples with SDS-NaOH at 65 °C for 30 min, while the enzymatic cleavage method involves incubating samples with Proteinase K at 56 °C for 2 h followed by 30 min heat inactivation. We applied these strategies to two purified rAAV vector stocks, rAAV2 and rAAV8, both pre-treated with Benzonase. As shown in Fig 4A, both methods resulted in comparable vg titer determinations across different batches. Notably, the SDS-NaOH method is advantageous due to its shorter processing time. Additionally, we assessed whether a single step of SDS-NaOH treatment under heat incubation was sufficient for the capsid dissociation of rAAV5, a serotype known for its high heat resistance among commonly used rAAV vectors. Fig 4B illustrates that there were no statistically significant differences in vg titers between rAAV2 and rAAV5 vectors across different incubation conditions. This finding indicates that the SDS-NaOH alkaline lysis method is effective for capsid dissociation across various rAAV serotypes, including the thermally stable rAAV5.

### 3. Inhibitory effects of the buffer components on qPCR reaction

**Fig 5:**
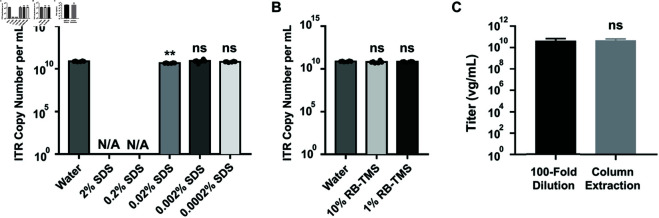
Comparison of Dilution method and Column DNA Extraction method before qPCR analysis. A and B: 1 *μ*g of plasmid pTR-UF11 was mixed with indicated buffer and subjected to qPCR assays. The data points utilized in Figures A and B for the control group are identical. SDS was normally introduced in rAAV vg titer sample for capsid dissociation. RB-TMS was normally introduced to resuspend freeze-thaw cells. C: ATCC AAV2 Reference Materials (rAAV2-RSS, ATCC VR-1616) were pre-digested by Benzonase and then incubated with SDS-NaOH. DNA isolations have been conducted 4 times. Before qPCR assay to determine vg titer, the samples were diluted 100-fold or subjected to DNA column extraction. Statistical analysis was performed by unpaired t-test. Titers were shown in the graph as Log10. Graph bars represent means ± SD of n = 4 or 6 experiments. ns: no significance, **: p ≤ 0.01.

To investigate the effects of buffer additives such as SDS (used in SDS-NaOH pretreatment) and RB-TMS (the cell resuspension buffer used in rAAV production), we performed parallel qPCR assays with varying concentrations of these components. In the experiments involving SDS, we observed that qPCR efficiency was completely inhibited by both 2% and 0.2% SDS, whereas concentrations of 0.002% SDS and below showed no significant difference compared to the ddH_2_O control group (Fig 5A). Notably, the 0.2% SDS concentration is equivalent to that used during SDS-NaOH treatment in rAAV vg titer determination. Additionally, qPCR assays revealed no significant differences among the groups with varying concentrations of RB-TMS (Fig 5B). Based on these results, we recommend diluting Benzonase & SDS-NaOH treated rAAV samples at least 100-fold for subsequent qPCR analysis.

To address whether extraction of vector DNA is necessary for quantifying rAAV vg titers, rAAV2 Reference Standard Stock (rAAV2 RSS) samples were pretreated with the Benzonase & SDS-NaOH, followed by either a 100-fold dilution with ddH_2_O or DNA extraction using a spin column kit (DNA Clean & Concentrator-25, ZYMO Research, US). The samples were then analyzed using qPCR assays to determine the rAAV vg titers. As illustrated in Fig 5C, the vg titers from both methods showed no significant variation and fell within the 95% confidence interval of the official rAAV2-RSS titer (2.70 × 10^10^ to 4.75 × 10^10^ vg/mL). In addition, the dilution method yielded vg titers of rAAV as reproducible as column extraction. Notably, the dilution method proved more advantageous due to its procedural simplicity.

### 4. Limit of detection and limit of quantidication of the Benzonase & SDS-NaOH method

**Fig 6:**
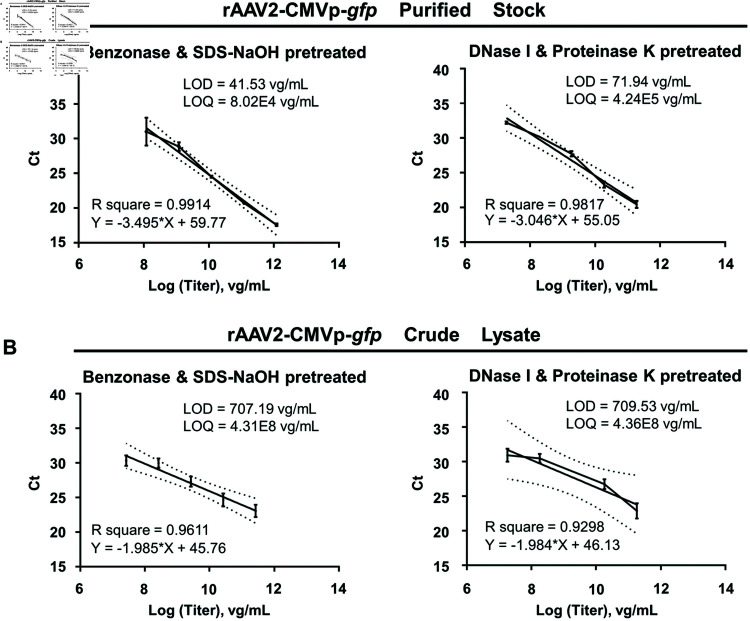
The LOD and LOQ for the sample pretreatment of VG titer utilizing qPCR assays. A series of 10-fold dilutions were employed to both crude rAAV2-CMVp-*gfp* vectors (A) and purified stocks (B). DNA isolations have been conducted 4 times. The Ct values were determined by qPCR following “Benzonase-SDS & NaOH" pretreatment (left) or “DNase I & Proteinase K" pretreatment (right). Titers were shown in the graph as Log10. Graph bars represent means ± SD of n = 5 or 7 experiments.

A series of 10-fold dilutions of both crude and purified rAAV2 samples was employed to establish the limit of detection (LOD) and limit of quantification (LOQ) for the qPCR assays. Prior to the qPCR analysis, all samples were subjected to either the “Benzonase & SDS-NaOH” pretreatment followed by a 100-fold dilution or the “DNase I & Proteinase K” treatment followed by purification using a DNA extraction spin column kit. Each set of regression curve data was obtained from a minimum of five replicates at each concentration level, with the limits of detection (LOD) and quantification (LOQ) calculated in accordance with ICH Q2(R1) guidelines [36]. For purified rAAV vector samples, those pre-treated with Benzonase and SDS-NaOH exhibited a LOD of 41.53 vg/mL and a LOQ of 8.02 × 10^4^ vg/mL, both of which were significantly lower than the LOD of 71.94 vg/mL and the LOQ of 4.24 × 10^5^ vg/mL recorded for samples pre-treated with DNase I and Proteinase K (Fig 6A). Similarly, crude lysates subjected to the same pre-treatment protocols demonstrated consistent results (Fig 6B), thereby highlighting the effectiveness of the former methodology in enhancing detection sensitivity during rAAV production. Subsequently, a series of 10-fold dilutions of the purified rAAV2 vectors were used to transduce HEK293 cells, where a 1:10,000 dilution corresponds to a dose of 2.7 vg/cell. The expression of the reporter gene was evaluated at 72 h post-transduction. To accurately quantify reporter gene expression, fluorescence intensity was measured using ImageJ software (S2A Fig) and a fluorescence-activated cell sorter (FACS) (S2B Fig). Both methods indicated a negative correlation between the fluorescence intensity and the 10-fold dilution series. At the highest dilution, minimal reporter gene expression was observed, suggesting that the “Benzonase & SDS-NaOH" pretreatment for detecting rAAV genome titers exhibits precise sensitivity and is robust against matrix interference.

### 5. Titration of Benzonase & SDS-NaOH pretreated rAAV vector samples by qPCR is applicable to different serotypes and reproducible across independent vector preparations

**Fig 7:**
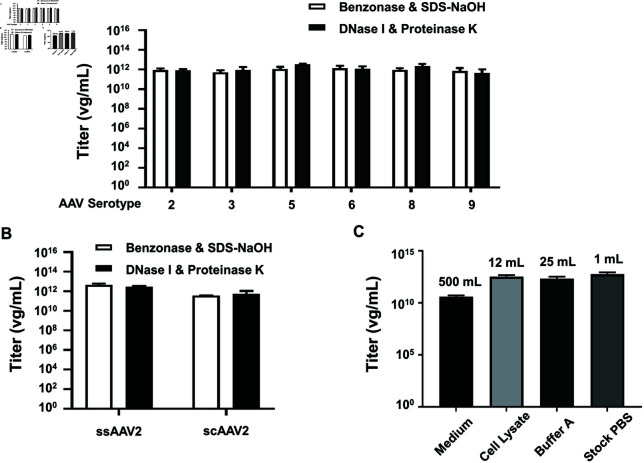
Comparisons of rAAV VG titers determined by “Benzonase & SDS-NaOH" or “Nase I & Proteinase K" pretreatment. Vg titers of rAAV2, 3, 5, 6, 8, 9-CMVp-*gfp* vectors (A) and ssAAV2 and scAAV2-CMVp-*gfp* vectors (B) were determined using either “Benzonase & SDS-NaOH" pretreatment (white bars) or “DNase I & Proteinase K" pretreatment (black bars). C: rAAV2-CMVp-*gfp* vectors at various production steps, such as cell medium, cell lysate, buffer A after iodixanol ultracentrifugation, and final purified stock PBS, were collected. The samples were subjected to “Benzonase & SDS-NaOH" pretreatment to determine the vg titers using qPCR assays. DNA isolations have been conducted 4 times. Titers were shown in the graph as Log10. Graph bars represent means±SD of n = 4 or 6 experiments.

To evaluate the applicability of the “Benzonase & SDS-NaOH” pretreatment method across different rAAV serotypes, a vg titration qPCR was conducted to evaluate a panel of rAAV serotypes, including rAAV2, 3, 5, 6, 8, and 9 296 (all expressing GFP). The samples treated with the “DNase I & Proteinase K" method were used as controls. As shown in Fig 7A, both pretreatment methods demonstrated robustness for all tested crude rAAV vectors. We also assessed the method’s applicability to both self-complementary AAV (scAAV) and single-stranded AAV (ssAAV) vectors (Fig 7B). Vg titration results indicated that ssAAV2 vectors exhibited approximately 10-fold higher titers than scAAV2 vectors, which aligns with previous reports [35]. To evaluate the production process of rAAV vectors, we quantified rAAV2 particles at different stages of production and purification, following the timeline described in the Methods and Materials Sect 2. The vg titers were measured for vector particles in the medium, crude cell lysate, Buffer A after gradient density centrifugation, and the final purified stock in PBS. The quantification revealed that HEK293 producer cells generated a substantial amount of vector particles in cell lysates (3.54 × 10^12^ ± 1.30 × 10^12^ vg/mL, total volume 12 mL) and released approximately 50% of the vector particles into the medium (4.19 × 10^10^ ± 1.18 × 10^10^ vg/mL, total volume 500 mL). During purification, the vector titer increased in Buffer A (2.14 × 10^12^ ± 1.27 × 10^12^ vg/mL, total volume 25 mL) and in the final stock PBS (5.96 × 10^12^ ± 2.72 × 10^12^ vg/mL, total volume 1 mL) due to the enrichment effect (Fig 7C). In summary, our findings demonstrate the versatility of the “Benzonase & SDS-NaOH" pretreatment for quantifying vg titers of various rAAV vectors, making it a valuable technique for titer detection throughout the rAAV production process.

## Discussion

In this study, the pretreatment “Benzonase & SDS-NaOH” approach has been implemented and validated for accurate quantification of rAAV vg via qPCR. The vg titration results showed a robust and efficient approach for digesting DNA/RNA residues and dissolving rAAV capsids to release vector DNA. This method enables time-efficient, less labor-intensive, and cost-effective analysis of rAAV vg titers [37]. As stated in the Introduction, a variety of methods have been developed for determining rAAV vg titers following sample pretreatment. For example, in Slot-blot assays, the released viral DNAs are typically applied to a nylon membrane, and probes are used to detect the vg titer of the rAAV sample. However, significant discrepancies in rAAV titers persist across various research laboratories, most of which employ the “DNase I & Proteinase K" pretreatment method for rAAV samples. This variability poses challenges for accurate comparison and evaluation of findings [38]. We hypothesize that the simple “Benzonase & SDS-NaOH" pretreatment may lead to more complete and consistent genome recovery, potentially reducing SDs among different laboratories worldwide, regardless of the methods used to quantify rAAV genomes [39]. Enhancing rAAV transduction efficiency and yield is a main objective of current research, with impacts on both the laboratory and industry, where monitoring production output is crucial. [40]. In addition to transient transfection [41], alternative methods have been employed for industrial rAAV production, including HeLaRC32 cells with replication-defective adenovirus transduction [42], herpes simplex virus infection (HSVi) [43], and Baculovirus expression vector system (BEVS) [44]. The producer cell lines (PCL), such as HeLaRC32, HEK293 and baby hamster kidney (BHK) cells, are all mammalian cells. On the other hand, in BEVS, insect Sf9 cells in Sf-900 II SFM with FBS are co-infected with three recombinant baculoviruses [45] and media in different manufacturing platforms may be compositionally diverse [46]. Detergents like Triton X-100 [47] are commonly used in industrial settings to lyse cells. Subsequently, unencapsulated nucleic acids are degraded using Benzonase [48], as described in this study. Clarification of cell culture lysates is achieved through filtration, typically involving single-stage filtration with 0.2 *μ*m membranes that require the addition of filter aids [49]. Whether the clarified suspension obtained in this step can be tested for AAV titer using our method merits further investigation. The next step involves removing contaminants such as cellular debris, proteins, and process-related impurities from clarified crude lysates, typically achieved through immunoaffinity chromatography (IC). IC introduces major ions like Na^+^ and Cl^-^ through binding and wash buffers, and high concentrations of chloride ions are known to interfere with qPCR assays [50], often necessitating sample dilution before analysis. Subsequently, rAAV full/empty particle separation is typically accomplished through ion exchange chromatography, consistent with methods described in this article. For the final step, tangential flow filtration (TFF) is widely used in the industry to concentrate viral particles, employing a neutral buffer with additives such as polysorbate 80 or Pluronic F68 [51]. These additives have been reported to mitigate their impact on qPCR assays through dilution [52]. The final concentrate can be stored in PBS. Overall, we hypothesize that our described method is suitable for process quality control in large-scale production, but further investigation is required. Other pivotal tools in gene therapy involve adenoviral (AdV) and retroviral vectors (RV), both of which are commonly produced in HEK293T cells. Crude vectors can be obtained through cell lysis and subsequently purified if necessary [53]. Accurate quantification of these viral vectors is crucial for ensuring their efficacy, safety, and consistency in gene therapy applications, as well as for monitoring the production and purification processes of viral particles [54]. The field of AdV titration encompasses both biological and physical titration methods. Biological titration methods, such as plaque assay (TClD50), cytopathic effect (CPE) assay, and fluorescence-activated cell sorting, assess the capacity of AdV to elicit cytopathic effects in transduced cells [55], Physical titration methods, including the high-performance liquid chromatography (HPLC) [56], UV spectrophotometry, Slot-blot analysis [57], ELISA [58], are typically used for purified samples. Recently, qPCR has become a standard technique for quantifying the genomic content of AdV, which is heavily dependent on effective sample pretreatment to remove unencapsidated nucleic acids and to release the viral DNA from the capsid [59]. Retroviral vectors, including lentiviral vectors, are enveloped viruses with single-stranded RNA genomes. The main challenge in quantifying these vectors is to efficiently extract the RNA and convert it into complementary DNA (cDNA) for accurate vg titration. This process necessitates the use of Benzonase, rather than DNase I, to degrade any encapsidated RNA or DNA, followed by the addition of detergents such as Triton X-100, SDS and sometimes proteinase to ensure thorough degradation. The suitability of the Benzonase & SDS-NaOH pretreatment for analyzing genome titers of various adenoviral and retroviral preparations, regardless of crude or purified, remains to be fully investigated. This pretreatment method introduces new possibilities for vg titer detection in crude or purified viral vectors. Its rapidity, simplicity, and reproducibility will support the design, refinement, and implementation of gene therapy not only based on rAAV, but also other viral vectors.

## Supporting information

S1 Appendix Original data and original gel images.
(ZIP)

S1 Table Materials and equipment required. (TIF)

S2 Table Composition of required solution in rAAV production and purification. (TIF)

S3 Table Composition of quantification PCR reaction mix. (TIF)

S4 Table Thermal cycling protocol for SYBR Green-based qPCR analysis. (TIF)

S1 Fig The efficiency of Benzonase and DNase I to digest nucleic acid impurities in ddH2O and crude cell lysate. (TIF)

S2 Fig Transduction efficiency of tested rAAV2-CMVp-gfp vectors in HEK293 cells. (TIF)
