# Supplementary figures and images for: An SDS-NaOH-based method to isolate genome of recombinant adeno-associated virus vectors for physical titer measurement
Source: PLoS One. 2025 Apr 3;20(4):e0315921. doi: 10.1371/journal.pone.0315921 (PMC11967980; doi:10.1371/journal.pone.0315921)

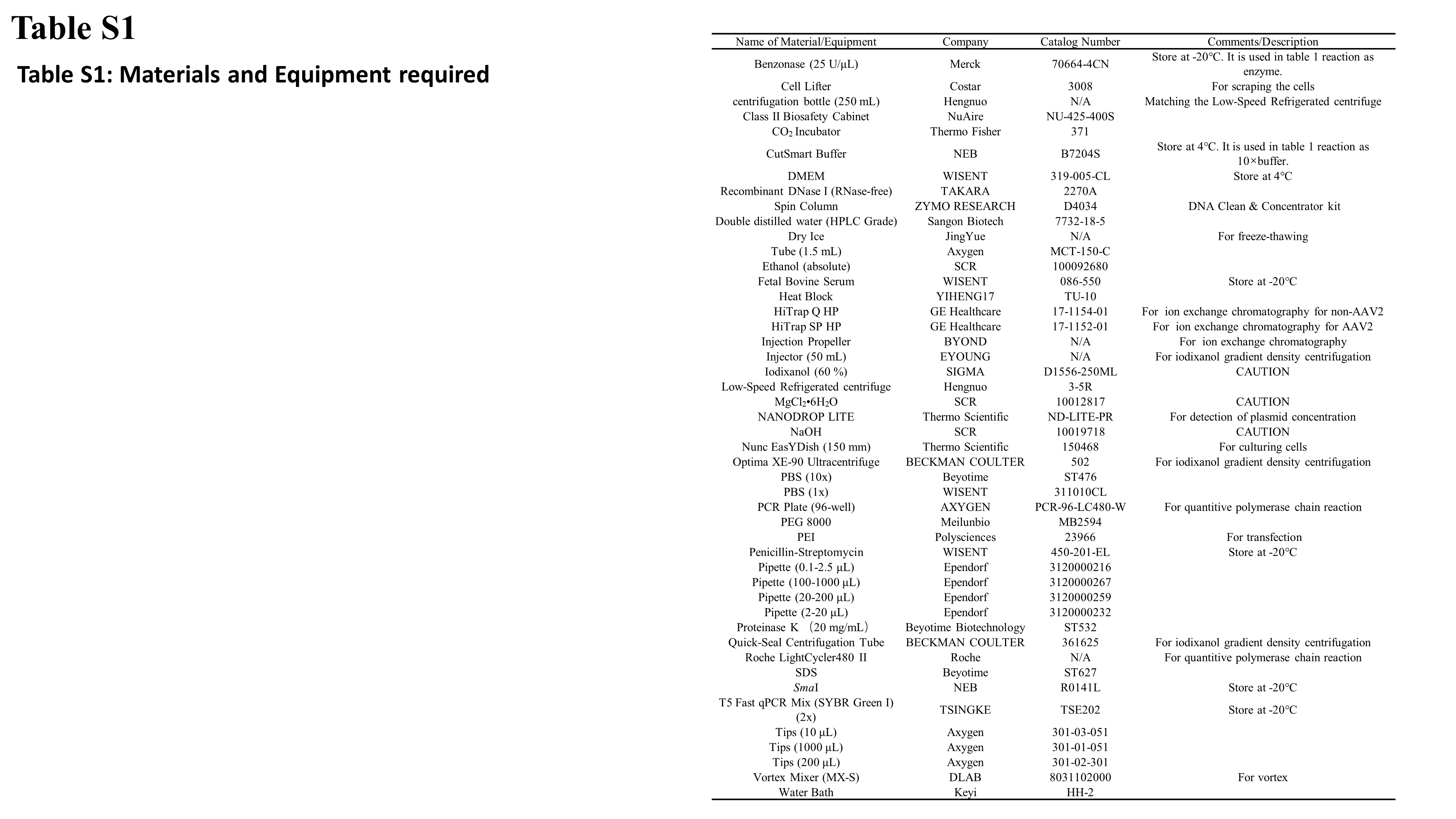

Supplement: S1 Table — (TIF) [file pone.0315921.s002.tif]

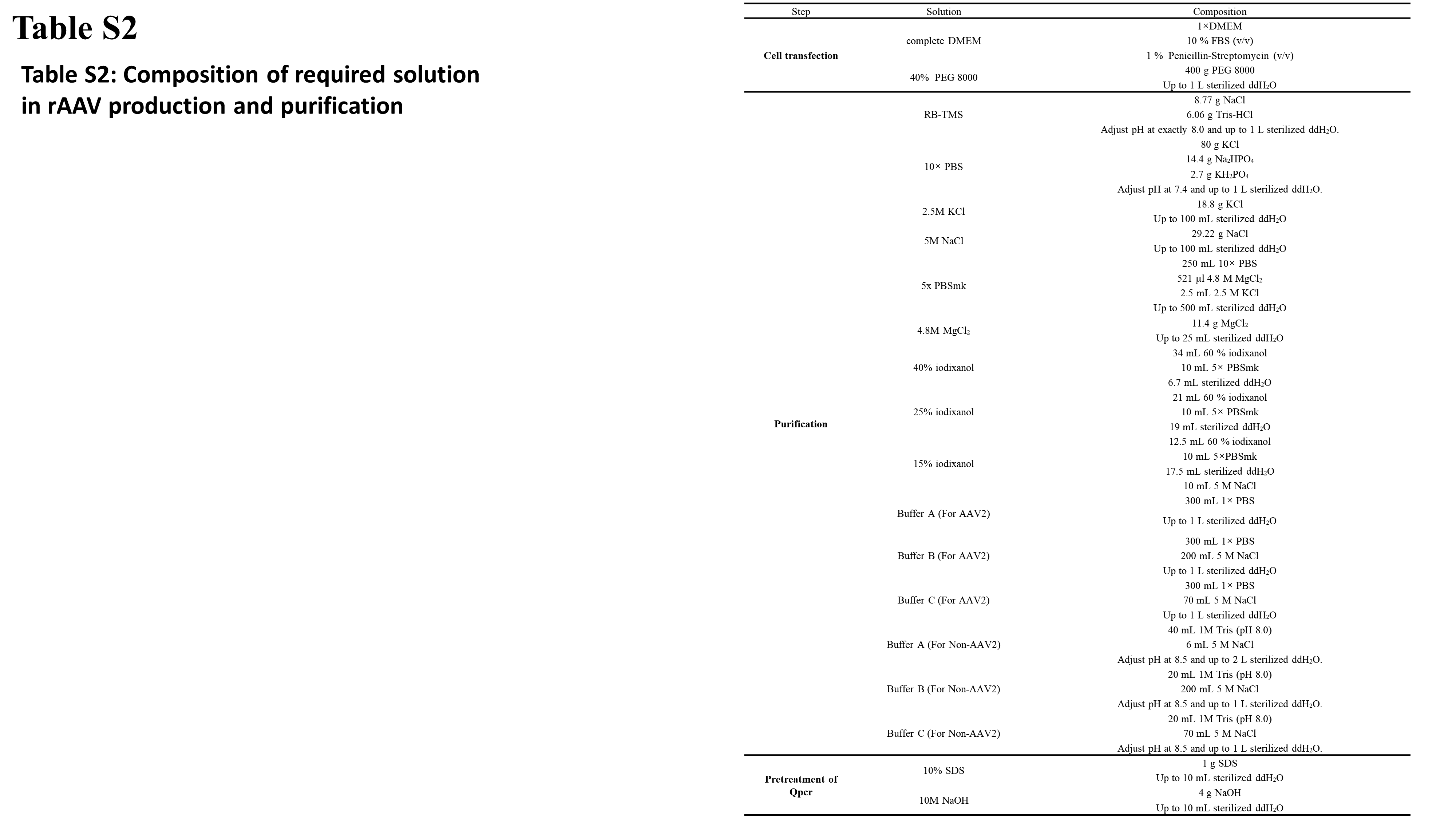

Supplement: S2 Table — (TIF) [file pone.0315921.s003.tif]

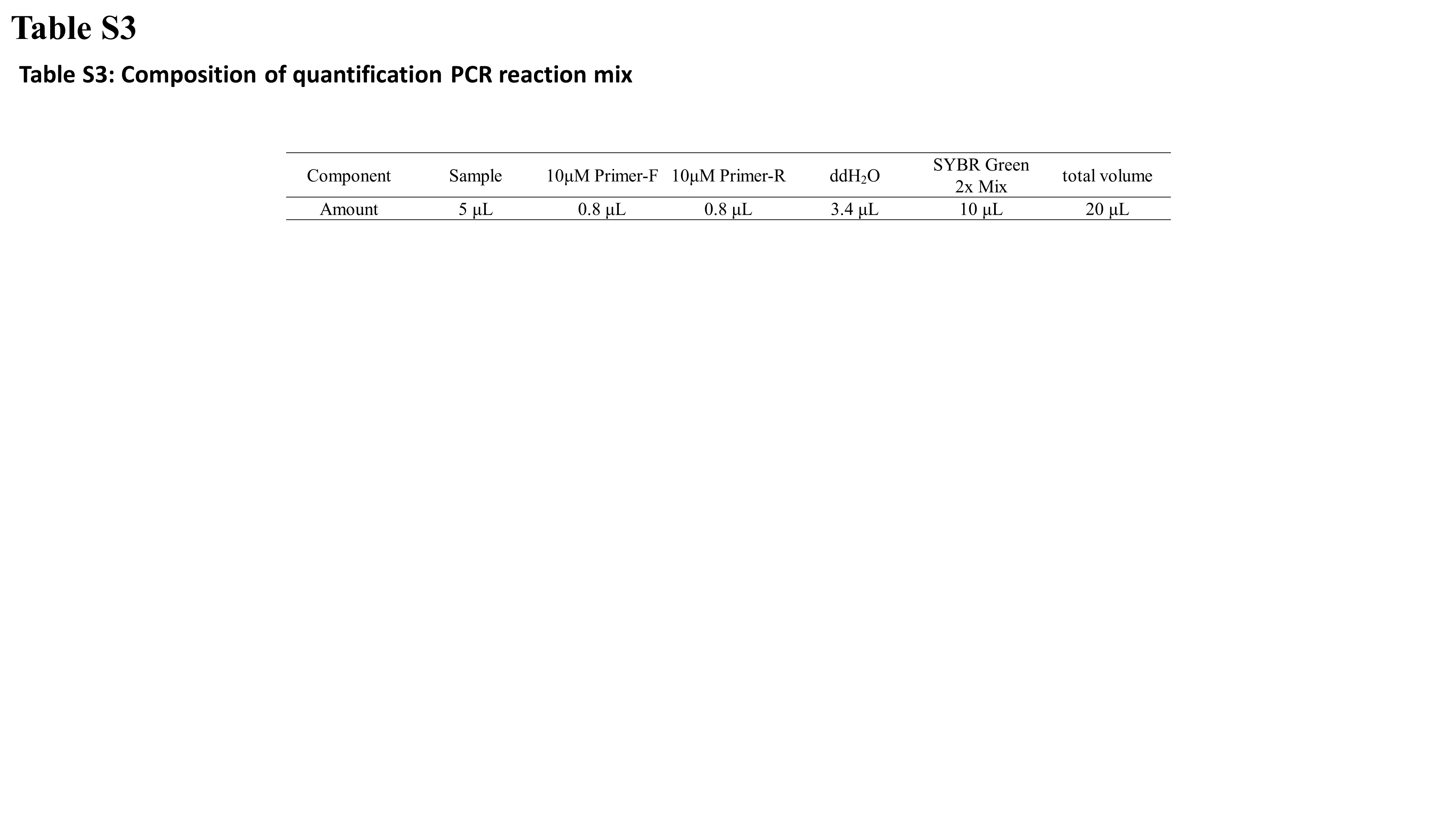

Supplement: S3 Table — (TIF) [file pone.0315921.s004.tif]

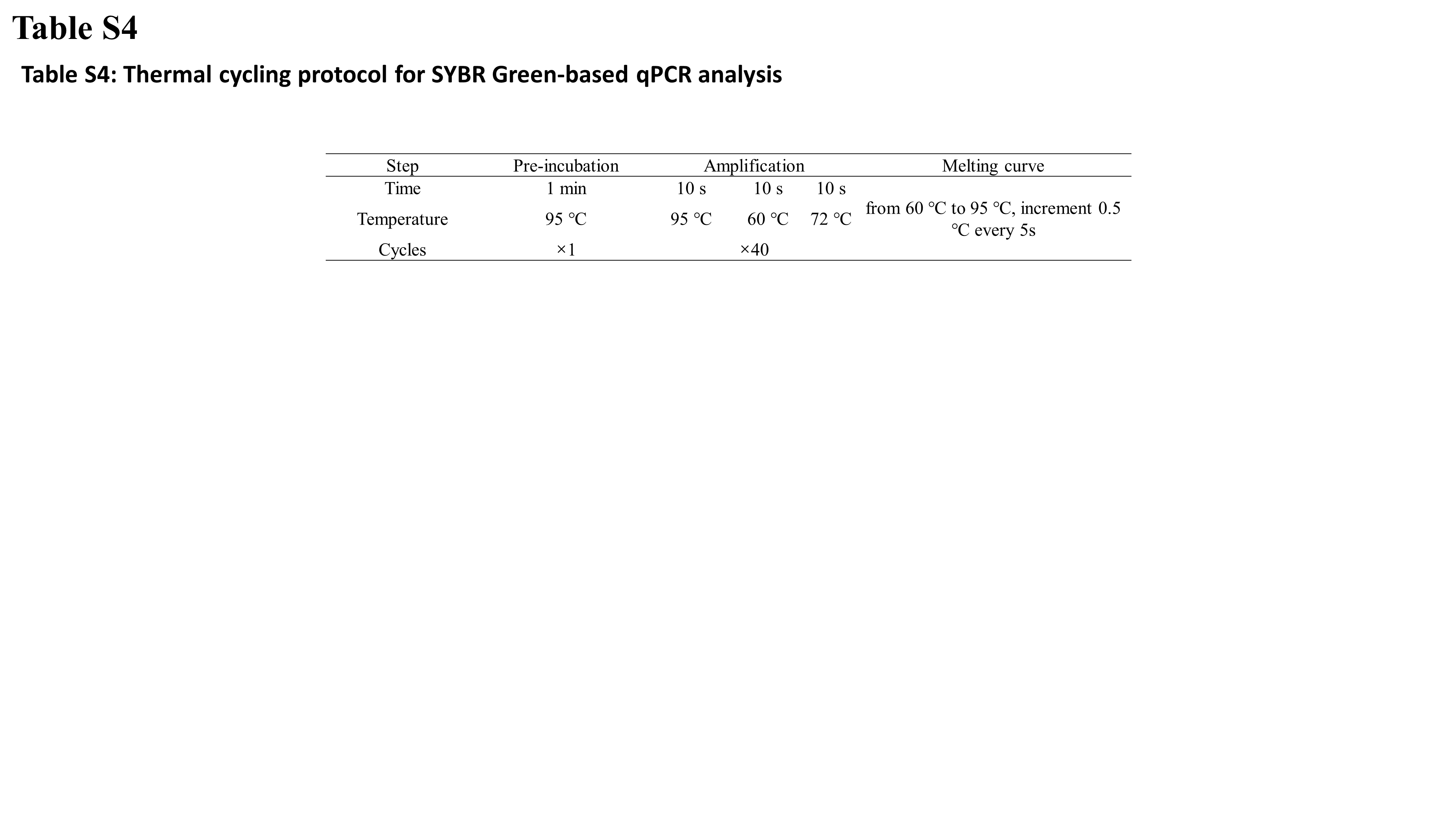

Supplement: S4 Table — (TIF) [file pone.0315921.s005.tif]

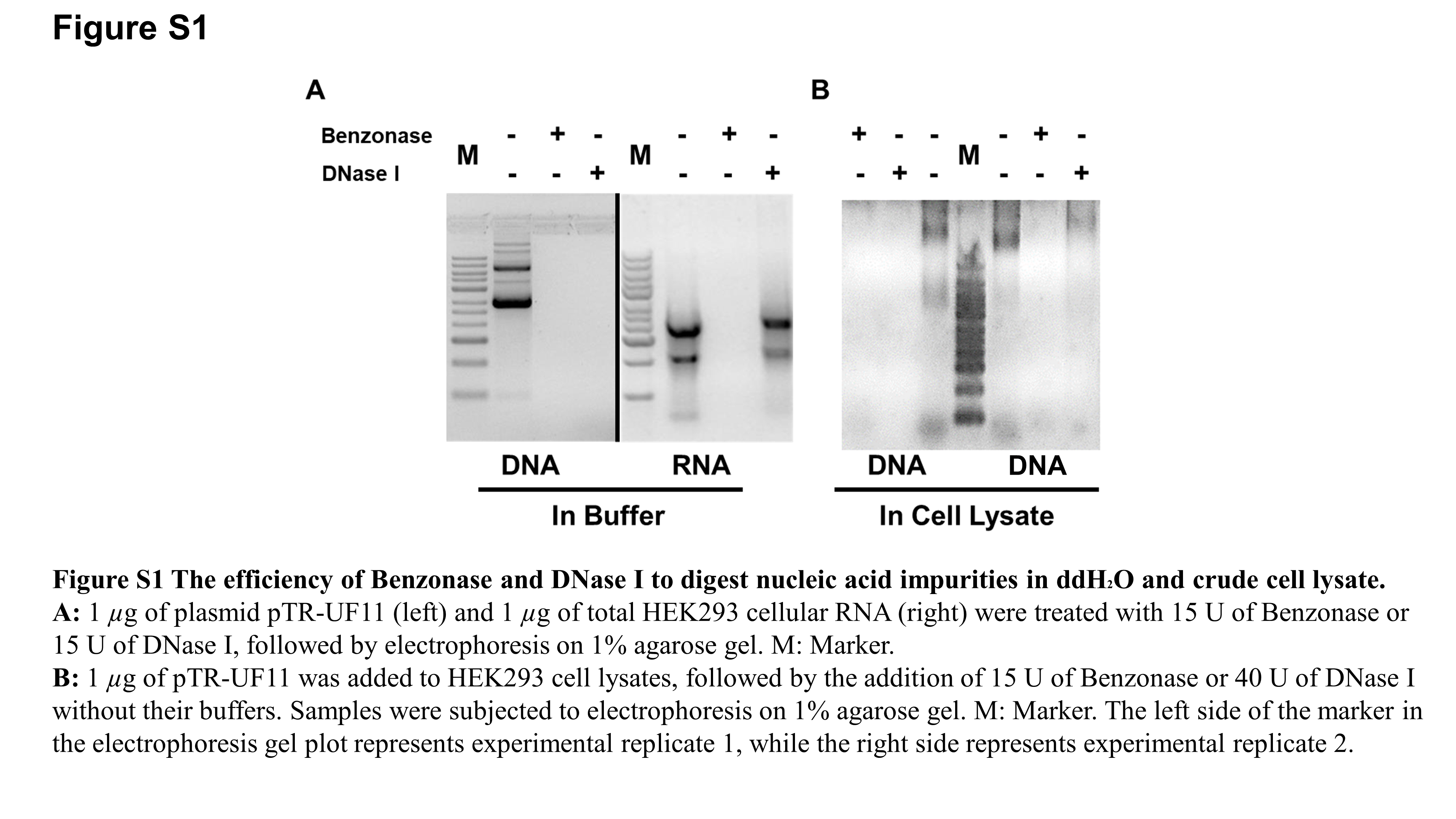

Supplement: S1 Fig — (TIF) [file pone.0315921.s006.tif]

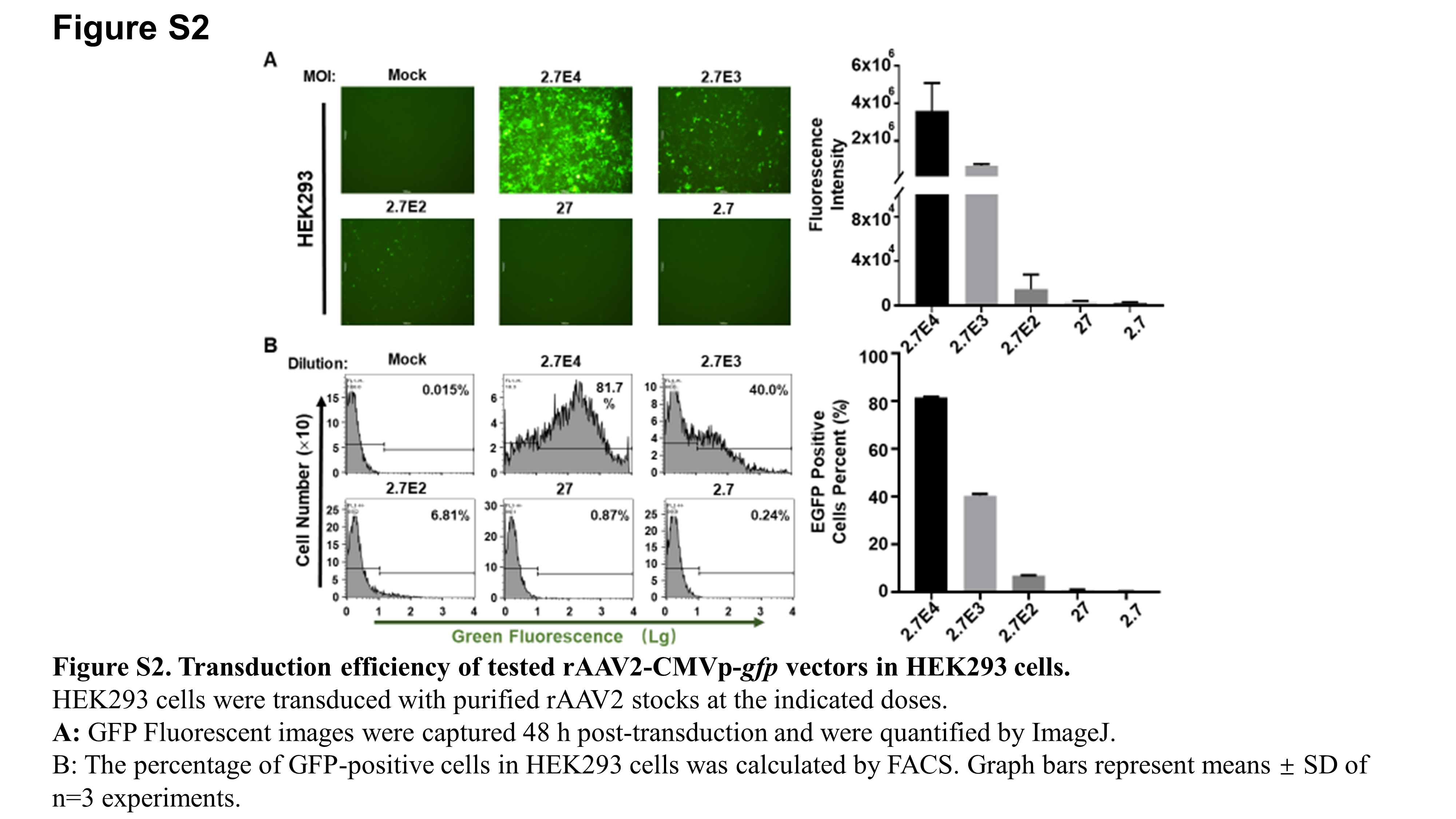

Supplement: S2 Fig — (TIF) [file pone.0315921.s007.tif]
